# Causal links of human serum metabolites on the risk of prostate cancer: insights from genome-wide Mendelian randomization, single-cell RNA sequencing, and metabolic pathway analysis

**DOI:** 10.3389/fendo.2024.1443330

**Published:** 2024-11-12

**Authors:** Renbing Pan, Jingwen Liu, Mingjia Xiao, Chuanyang Sun, Jianyong Zhu, Lijun Wan, Boxin Xue

**Affiliations:** ^1^ Department of Urology, The Second Affiliated Hospital of Soochow University, Suzhou, Jiangsu, China; ^2^ Department of Urology, The Quzhou Affiliated Hospital of Wenzhou Medical University, Quzhou People’s Hospital, Quzhou, Zhejiang, China; ^3^ The Quzhou Affiliated Hospital of Wenzhou Medical University, Quzhou People’s Hospital, Quzhou, Zhejiang, China; ^4^ Department of Central Laboratory, The Quzhou Affiliated Hospital of Wenzhou Medical University, Quzhou People’s Hospital, Quzhou, Zhejiang, China

**Keywords:** serum metabolites, prostate cancer, Mendelian randomization, single-cell RNA-seq, metabolic pathways

## Abstract

**Background:**

Recently, serum metabolites have shown potential in predicting survival outcomes and may be related to the pathogenesis of prostate cancer. Nevertheless, the precise impact concerning the genetic effect of metabolites on prostate cancer risk remains obscure. In this context, we conducted a Mendelian randomization (MR) study aiming to explore the causality between genetically determined metabolites and the risk of prostate cancer.

**Methods:**

We conducted a two-sample MR analysis aiming to identify the underlying metabolites associated with prostate cancer. Exposure information was obtained from the largest metabolome-based genome-wide association (GWAS) data containing 7,824 Europeans. Genome-wide association analysis was utilized to detect instrumental variables (IVs) for metabolites. We applied the inverse-variance weighted (IVW) approach as the primary method, and to augment the reliability and robustness of our findings, additional analysis methods encompassing weighted median, MR-Egger, and leave-one-out analysis were utilized. MR-Egger intercept test was implemented to explore the pleiotropy. Cochran’s Q test was utilized to quantify the degree of heterogeneity. Additionally, we performed metabolic pathway analysis and single-cell RNA sequencing analysis.

**Results:**

We found that three serum metabolites were causally associated with prostate cancer after utilizing rigorous screening standards. Utilizing single nucleotide polymorphisms as IVs, a 1-SD increase in fructose was associated with 77% higher risk of prostate cancer (OR:1.77, 95%CI: 1.05-2.97, P_IVW_=0.031), a 1-SD increase in N1-methyl-3-pyridone-4-carboxamide was associated with 29% higher risk of prostate cancer (OR:1.29, 95%CI: 1.05-1.58, P_IVW_=0.017), and a 1-SD increase in 12-hydroxyeicosatetraenoate (12-HETE) was associated with 18% higher risk of prostate cancer (OR:1.18, 95%CI: 1.07-1.31, P_IVW_=0.0008). Metabolites that were causally linked to the risk of prostate cancer were mainly enriched in the valine, leucine and isoleucine biosynthesis pathway (P=0.026) and the nicotinate and nicotinamide metabolism pathway (P=0.048).

**Conclusions:**

Our MR analysis provided suggestive evidence supporting the causal relationships between three identified serum metabolites and prostate cancer, necessitating further investigation to elucidate the underlying mechanisms through which these blood metabolites and metabolic pathways may impact the initiation and progression of prostate cancer.

## 1 Introduction

Globally, prostate cancer is the second most common malignancy of male cancer, with an incidence rate second only to lung cancer, affecting millions of men worldwide (1). In 2020, there were an estimated 1.4 million newly diagnosed cases of prostate cancer, resulting in approximately 375,000 fatalities (2). The growth of prostate cancer cells is dependent on androgen, so androgen deprivation therapy (ADT) is an effective therapeutic strategy widely used in clinical practice (3). Additionally, the etiology of prostate cancer remains largely unknown, although certain risk factors have been identified, encompassing advanced age, baldness, genetic mutations, and positive family history (4).

To our knowledge, the identification of the risk factors of prostate cancer plays a pivotal role in disease prevention and the establishment of early screening protocols. Previous studies have indicated that several modifying factors, such as smoking, obesity, and nutritional and metabolic factors, may contribute to an increased risk of prostate cancer (5). A previous study on biomarkers conducted by Couzin J et al. revealed that metabolite in urine may point to high-risk prostate cancer (6). Meanwhile, Kosti O et al. observed subtle disparities in estrogen metabolite concentrations between prostate cancer patients and non-cancer individuals, indicating that estrogen metabolites could serve as independent biomarkers for assessing the risk of prostate cancer (7). Currently, accumulating evidence has indicated that diverse blood metabolites might be related to prostate cancer biological processes. Previous studies performed by Ostman JR et al. suggested that alterations in plasma concentrations of metabolites involved in lipid, aromatic amino acid, and glucose metabolism were correlated with the risk of developing prostate cancer (8). In addition, another research conducted by Cardoso HJ et al. demonstrated that the acceleration of prostate cancer cell survival and growth was mainly attributed to the sustainability of glucose, lipids, and glutamine (9). Interestingly, another study showed that blood methionine metabolites were identified as a significant risk factor for the progression of metastatic prostate cancer (10). In contrast, a previous study conducted by O’Flaherty JT et al. demonstrated that the metabolites of docosahexaenoic acid produced by 15-lipoxygenase inhibit the proliferation and survival of prostate cancer cells (11). Taken together, considering the susceptibility of observational studies to underlying control bias and reversed causality (12), further investigation into the potential causal links between genetically determined human serum metabolites and prostate cancer was still required.

Performing randomized controlled trials (RCTs) to explore the influence of human serum metabolites on the risk of prostate cancer is impractical due to ethical considerations. In addition, the causal nature of the associations between blood metabolites and prostate cancer remains obscure in observational research due to underlying residual confounders and reverse causality issues. Thus, alternative approaches that facilitate casual inference can provide valuable insights into whether specific metabolites represent potentially risky factors. Mendelian randomization (MR) is a novel epidemiology technique that leverages genetic effects to evaluate the inference of a causal relationship between an exposure and an outcome (13). Conceptually, MR shares similarities with RCTs as genetic variants are randomly assigned during meiosis, thereby reducing concerns related to the confounders and reverse causality (14).

A previous meta-analysis of genome-wide association study (GWAS) was performed by Shin. et al. to explore the genetic basis for 486 serum metabolites (15), providing a dataset to detect the underlying causality with some diseases related to metabolic factors. For instance, a previous MR study showed that genetically determined levels of specific serum metabolites exhibited causal effects on the occurrence and progress indicators of chronic kidney disease (12). Furthermore, another MR study suggested that targeted interventions of specific blood metabolites could mitigate the risk of sarcopenia (16). As of now, there is still limited research on the correlation between blood metabolites and the risk of prostate cancer. In addition, the causality between blood metabolites and prostate cancer has yet to be established through MR analysis. Thus, in this study, by conducting a two-sample MR and metabolic pathways analysis, we would systematically evaluate the potential causal links between serum metabolites and the risk of prostate cancer. Our findings will provide vital implications for a better understanding of the correlation between serum metabolites and the initiation and progression of prostate cancer. Elucidating these causal links will provide greater biological insight and explain the mechanism for prostate cancer.

## 2 Materials and methods

### 2.1 Study design

**Figure 1:**
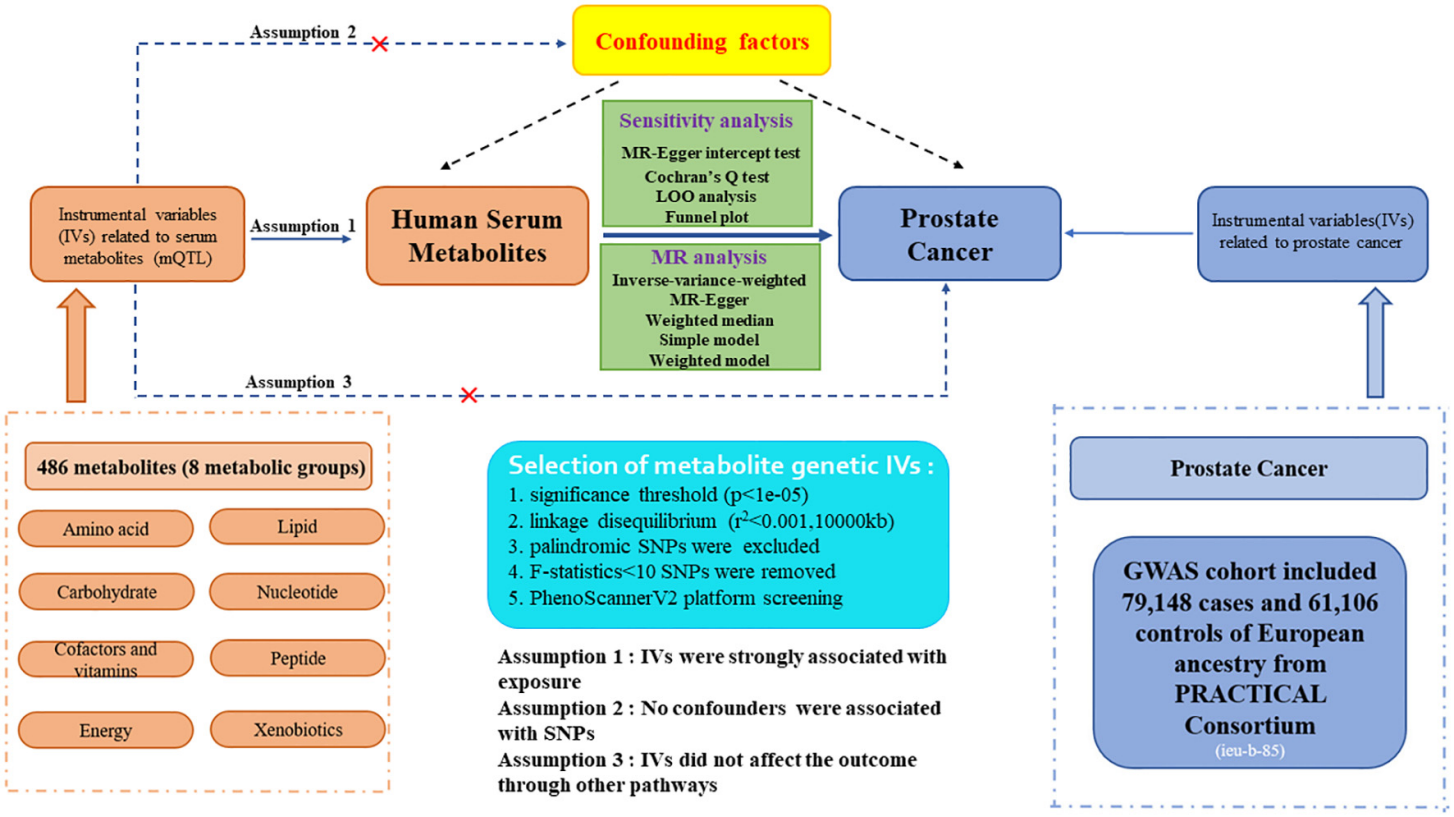
Study design and detailed flowchart of two-sample Mendelian randomization for 486 human serum metabolites and prostate cancer. IVs, instrumental variables; mQTL, metabolites quantitative trait loci; SNP, single nucleotide polymorphism.

The flow diagram and design for this study is displayed in **Figure 1**. We performed a two-sample MR analysis to estimate the causal links between 486 human blood metabolites and prostate cancer utilizing GWAS summary statistics, with detailed characteristics and information listed in **Supplementary Table S1**. The detailed steps included the following three steps: (1) eligible single nucleotide polymorphisms (SNPs) associated with 486 blood metabolites were extracted according to the specified threshold criteria. (2) the two-sample MR method was utilized to analyze the relationships between serum metabolites and prostate cancer one by one. (3) sensitivity analysis was performed on the findings of MR estimates. To augment the robustness and stability of the MR analysis results, instrumental variables (IVs) were extracted to satisfy the three core assumptions. Firstly, the IVs must be strongly associated with human serum metabolites. Secondly, no confounding factors are related to the IVs. Thirdly, the IVs affect the outcome only via exposure and there are no other causal pathways for the IVs to impact the outcome (17). We selected genetic IVs for each human serum metabolite to investigate the causal association of each serum metabolite to prostate cancer. The utilization of publicly attainable GWAS summary datasets obviated the requirement for ethical approval. All of the MR analysis and sensitivity analysis programs were conducted with the R package “Two Sample MR” (version 4.2.3).

### 2.2 Data sources

#### 2.2.1 Human serum metabolites

The metabolite database was acquired from one of the most extensive metabolite studies by Shin et al (15). The human serum metabolites in the whole genome-wide association study (GWAS) data were from the Metabolomics GWAS server (https://metabolomics.helmholtz-muenchen.de/gwas/). Our study contained a total of 7824 participants of European ancestry, which included 1768 from the KORA F4 study in Germany and 6056 from the UK Twin Study, and approximately 2.1 million SNPs for 486 metabolites were tested. Among the 486 serum metabolites, 177 metabolites were defined as unknown. Additionally, another 309 metabolites were categorized chemically and allocated to eight broad metabolic groups, encompassing amino acids, peptides, lipids, cofactors and vitamins, carbohydrates, energy, nucleotides, and xenobiotic metabolism, as documented in the Kyoto Encyclopedia of Genes and Genomes (KEGG) database (18). The detailed information and characteristics of the 486 serum metabolites are presented in **Supplementary Table S2**.

#### 2.2.2 Prostate cancer

The summary datasets for prostate cancer were acquired from the largest GWAS meta-analysis, which included 79,148 cases and 61,106 controls of European ancestry from the PRACTICAL consortium (19). For each GWAS, the analysis was adjusted for both specific principal components and relevant covariates, while the overall meta-analysis took into consideration the influence of principal components. This meta-analysis datasets contain 20,346,368 single nucleotide polymorphisms (SNPs). In this consortium, multiple cohort studies relied upon prostate cancer and mortality registry as well as other data for the endpoints. All classification schemes incorporated the diagnostic clinical features PSA, tumor stage, and Gleason score.

### 2.3 Selection criteria for instrumental variables

The eligible genetic instrumental variables associated with human serum metabolites were selected through a series of control steps. Firstly, given the scarcity of SNPs reaching genome-wide significance, we broaden the threshold value to *P*<1e-05 to extract qualified IVs, consistent with Chang X. et al.’s study (20). Finally, all 486 serum metabolites were identified under this criterion. Secondly, the influence of robust linkage disequilibrium (LD) between SNPs was mitigated by employing an LD cutoff for the extracted SNPs (r²<0.001, 10,000kb windows), ensuring independence among IVs for each exposure (21). Thirdly, the palindromic SNPs with intermediate allele frequency were excluded from the GWAS of human serum metabolites due to the absence of provided allele frequencies. Fourthly, to eliminate bias arising from weak IVs, we calculated the F-statistics for each SNP to assess statistical strength. The F-statistic was used to assess the strength of each SNP as an instrumental variable, with a value exceeding 10 indicating a robust instrument (22). The F-statistic is a statistic method that captures the magnitude and accuracy of the genetic effect on the trait. It can be calculated as F=R^2^(N-2)/(1-R^2^), where R^2^ represents the proportion of variance in the trait illuminated by the SNP, and N denotes the sample size of GWAS involving SNPs with the trait (23). Moreover, the outcome-related SNPs (*P*-value<1e-05) were excluded. The brief selection standard of metabolites genetic IVs in this study is presented in **Figure 1**.

### 2.4 Statistical analysis

To identify the causal effects of human serum metabolites linked to the risk of prostate cancer by conjoining various SNPs, we conducted a two-sample Mendelian randomization analysis utilizing five analysis models. The dominant approach we employed was the standard inverse variance weighted (IVW) estimates, which combined the Wald ratio estimation of each SNP and demonstrated superior statistical power compared to other diverse MR methods (24). This approach was regarded as the primary method of assessing the potential causal relationships between genetically predicted serum metabolites and prostate cancer. The analysis we conducted required data on SNPs, alleles, effect sizes, *P*-values, and allele frequencies (EAF) (25). Additionally, other MR analysis approaches, encompassing MR-Egger, weighted median, simple mode, and weighted mode were implemented as auxiliary analysis methods to IVW by enhancing the robustness of estimates and expanding their applicability in various scenarios (26). The MR-Egger method is capable of detecting violations of the IVs assumption and providing consistent estimates even when using invalid instruments (27). We applied additional analyses of MR approaches with diverse modeling assumptions and strengths (weighted median, simple mode, and weighted mode) to augment the robustness and stability of the findings.

### 2.5 Sensitivity analysis

To validate that IV affects the outcome solely through the exposure, and to augment the reliability and strength of the results, various sensitivity analyses were also needed. Thus, diverse methods including MR-Egger intercept test, Cochran’s Q test, funnel plot, and leave-one-out (LOO) analysis were employed to validate the robustness of the significant findings (P_IVW_<0.05) (28). Among them, the MR-Egger intercept test was performed to identify the existence of horizontal pleiotropy, where statistical significance was determined by *P*-values for the intercept below 0.05 (29). Moreover, Cochran’s Q test was employed to assess heterogeneity among SNPs in both IVW and MR-Egger analyses. A *P*-value exceeding 0.05 in Cochran’s Q statistics indicated the absence of heterogeneity among SNPs (30). Finally, we applied LOO analysis to explore whether results were influenced by individual SNP (31). In addition, we also conducted the MR Steiger directionality test to ensure whether our findings supported our hypothesis (32). Eventually, the Bonferroni correction was applied to address the issue of multiple comparisons, and a significance level of *P*<1.03e-04 (0.05/486) was adopted (Bonferroni correction with 486 tests) (12). We also reported metabolites that exhibited a *P*-value less than 0.05, but exceeded the Bonferroni-corrected threshold, indicating suggestively causal associations with prostate cancer. Thus, underlying eligible candidate metabolites for participation in the risk of prostate cancer were identified by multiple standards: (1) *P*-value for the dominant MR analysis was significant (P_IVW_<0.05). (2) There should be at least one other method with a *P*-value<0.05 except the IVW approach. Furthermore, consistent direction and magnitude among the five approaches (scatter plots). (3) No heterogeneity or horizontal pleiotropy was explored. (4) The LOO analysis did not identify any significant influential points.

### 2.6 Confounding analysis

Although we assessed the heterogeneity and horizontal pleiotropy of the Mendelian randomization (MR) results through multiple sensitivity analysis approaches to explore any SNPs that violated Assumptions 2 and 3 of MR (**Figure 1**), there may also be little residual confounding IVs. Therefore, to fulfill the second assumption of the MR study, SNPs related to confounding factors, encompassing blood pressure, body mass index (BMI), and smoking, were removed by the PhenoScannerV2 website tool (http://www.phenoscanner.medschl.cam.ac.uk/) screening. If any SNP was found to be associated with these confounders (*P*<1e-05), MR analysis would be re-conducted after excluding this SNP to confirm the robustness of the findings. In addition, the GeneMANIA database was utilized to perform a protein-protein interactive (PPI) network on gene and protein pathways, co-localization, co-expression, and functional assays with pinpoint accuracy of prediction algorithm (33).

### 2.7 Single-cell RNA sequencing data analysis

We obtain the single-cell RNA-sequencing (scRNA-seq) data of prostate tissue from the Panglao DB database (https://panglaodb.se/), a user-centric single-cell sequencing dataset for the scientific community that focused on single-cell RNA sequencing tests from mice and humans (34). We utilized the “Sample” module to retrieve the datasets. “human” and “tissue” were set as filter protocols. The “Seurat” R package was employed to analyze the scRNA-seq data (35). This database encompassed 1368 single-cell RNA-sequencing dataset samples. The publicly attainable datasets utilized in this study had acquired the essential ethical approvals.

### 2.8 Metabolic pathways and enrichment analysis

The chosen metabolite metabolic pathway and enrichment analysis were explored using the MetaboAnalyst6.0 platform (http://dev.metaboanalyst.ca/) (36). Functional enrichment analysis and the metabolic pathway module were utilized to identify potential metabolite groups or pathways that may be related to the occurrence and progression of prostate cancer. The metabolite databases, encompassing the Small Molecule Pathway Database (SMPDB) and the KEGG database, were employed in our study. In particular, only known serum metabolites exceeding the recommended threshold (P_IVW_<0.05) were analyzed for metabolic pathways.

## 3 Results

### 3.1 Selection of the instrumental variables

We performed a two-sample MR analysis to estimate the causal effects of genetically predicted metabolites on the risk of prostate cancer. Taking account of the limited genetic variance, as well as the restricted number of SNPs and low statistical powers, the MR analysis was performed by broadening the cutoff to *P*-value<1e-05. The number of identified IVs for 486 metabolites ranged from 3 to 478, with a median number of 15 (**Supplementary Table S2**). Furthermore, the minimum F-statistics of these IVs was 17.84, indicating that all IVs were sufficiently effective for the MR analysis and that the weak instrumental bias was improbable to happen (all F-statistics >10).

### 3.2 Causal associations of genetically predicted metabolites on prostate cancer

**Figure 2:**
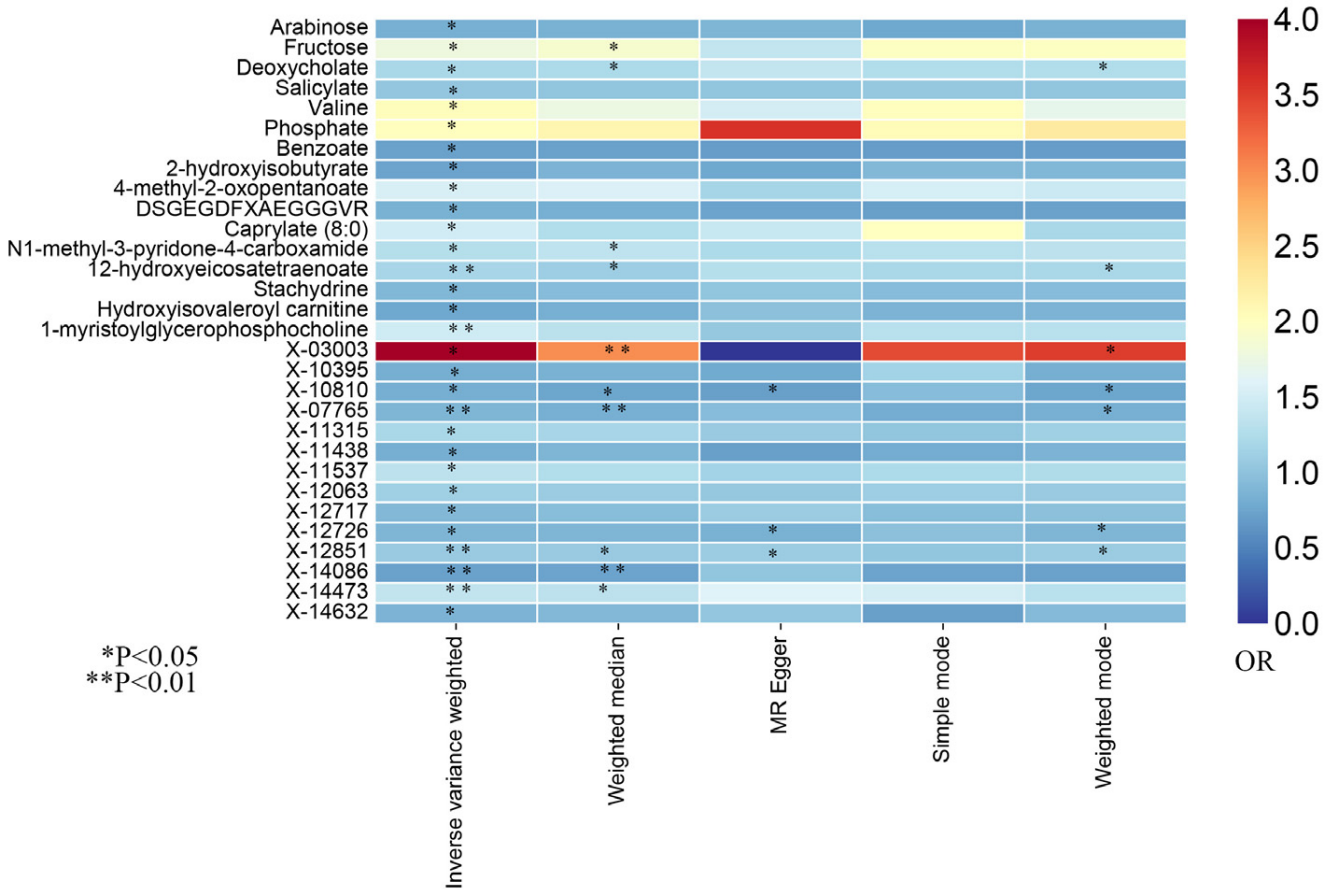
The heatmaps of five Mendelian randomization analysis methods. Different color blocks represent different odds ratio values. OR, odds ratio.

In this study, the IVW approach was utilized as the dominant approach in assessing the causal links between 486 serum metabolites and the risk of prostate cancer. Despite the Bonferroni correction yielding no significant causal relationships of serum metabolites with prostate cancer, a total of 30 blood metabolites, including 16 known metabolites and 14 unknown metabolites, exhibited suggestive associations with prostate cancer at the standard significance level of 0.05 (P_IVW_<0.05). Moreover, the MR-Egger, weighted mode, simple mode, and weighted median methods consistently yield causal estimates that are consistent in terms of both direction and magnitude. To enhance the visual representation of IVs’ strength and facilitate a more intuitive understanding of the compiled data, we utilized heatmaps to present this aspect of the information (**Figure 2**). The explicit MR estimates of diverse approaches were presented in **Supplementary Table S3**.

### 3.3 Sensitivity analysis

Due to the susceptibility of IVW approaches to weak instrumental bias, the implementation of sensitivity analyses was essential to ensure the robustness and stability of the causal relationship. **Supplementary Table S4** showed the sensitivity analysis results for evaluating the reliability of our MR estimates. MR-Egger intercept test manifested no significant pleiotropy except only X-14632 (P _Egger-intercept_=0.02). Furthermore, MR-Egger and IVW Cochran’s Q tests were applied to explore the heterogeneity. There was no indication of significant heterogeneity among SNPs for those identified metabolites except deoxycholate, salicylate, caprylate (8:0), X-03003, X-11438, and X-12851 (Q-P_IVW_, Q-P_MR-Egger_, and P _Egger-intercept_ as shown in **Supplementary Table S4**).

### 3.4 Identification of specific serum metabolites linked to the risk of prostate cancer

**Figure 3:**
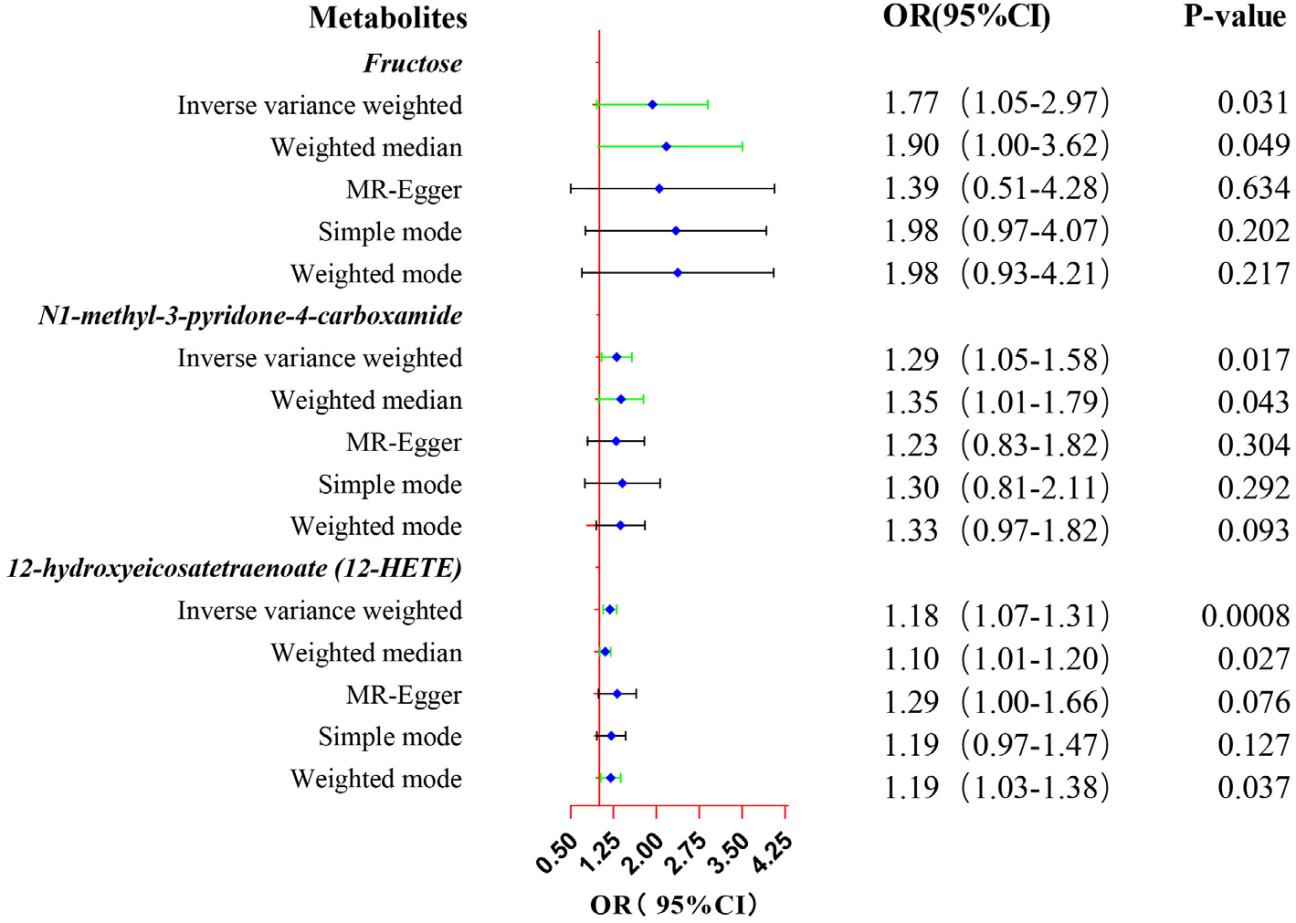
Forest plot of the MR estimates for the associations between three identified metabolites and prostate cancer. The inverse variance weighted method is considered the main method. CI, confidence interval; OR, odds ratio.

**Figure 4:**
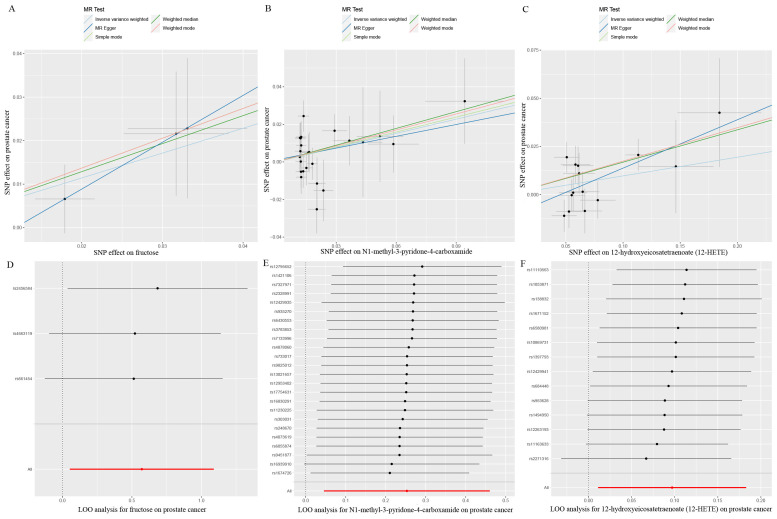
Scatter plots and LOO sensitivity analysis results of MR analysis between three identified metabolites and prostate cancer. **(A, D)** fructose, **(B, E)** N1-methyl-3-pyridone-4-carboxamide, **(C, F)** 12-hydroxyeicosatetraenoate (12-HETE). SNP, single nucleotide polymorphism.

Eventually, after conducting comprehensive complementary and sensitivity analyses, three known metabolites that have been extensively screened were identified as significantly eligible candidates linked to the risk of prostate cancer. Specifically, as shown in **Figure 3**, fructose (OR:1.77, 95%CI: 1.05-2.97, P_IVW_=0.031), N1-methyl-3-pyridone-4-carboxamide (OR:1.29, 95%CI: 1.05-1.58, P_IVW_=0.017), and 12-hydroxyeicosatetraenoate (12-HETE) (OR:1.18, 95%CI: 1.07-1.31, P_IVW_=0.0008) were identified as the most significant risk factors for both the occurrence and progression of prostate cancer. Additionally, scatter plots across diverse tests of links of three genetically determined metabolites with the risk of prostate cancer were displayed in **Figures 4A–C**. The results of the LOO analysis similarly indicated that none of the individual SNPs exerted a significant impact on the robustness of analysis outcomes (**Figures 4D–F**). To sum up, scatter plots and LOO analysis showed that the findings were not affected by outliers. In addition, **Table 1** presented the sensitivity analysis findings, indicating the reliability and stability of our MR analysis findings (all *P*-values>0.05). Moreover, as shown in the funnel plots (**Supplementary Figure S1**), the distribution of SNPs was symmetrical, suggesting that estimates were not violated. Lastly, the findings demonstrated that the *P*-values of three identified metabolites were all between 1.03e-04 and 0.05, indicating that these serum metabolites were suggestively associated with prostate cancer. Further studies are required to validate their correlation in the future.

**Table 1:** Horizontal pleiotropy and heterogeneity analysis for the three identified metabolites.

Metabolite	Subcategory	Pleiotropy		Heterogeneity			
		MR-Egger Intercept test		MR-Egger		IVW	
		Intercept	*P-*value	Q-statistic	*P-*value	Q-statistic	*P-*value
Fructose	Carbohydrate	-0.0127	0.669	0.0002	0.989	0.329	0.849
N1-methyl-3-pyridone-4-carboxamide	Nucleotide	0.0011	0.798	25.769	0.262	25.847	0.308
12-hydroxyeicosatetraenoate(12-HETE)	Lipid	-0.0114	0.232	15.332	0.224	17.361	0.183

### 3.5 Confounding analysis

**Figure 5:**
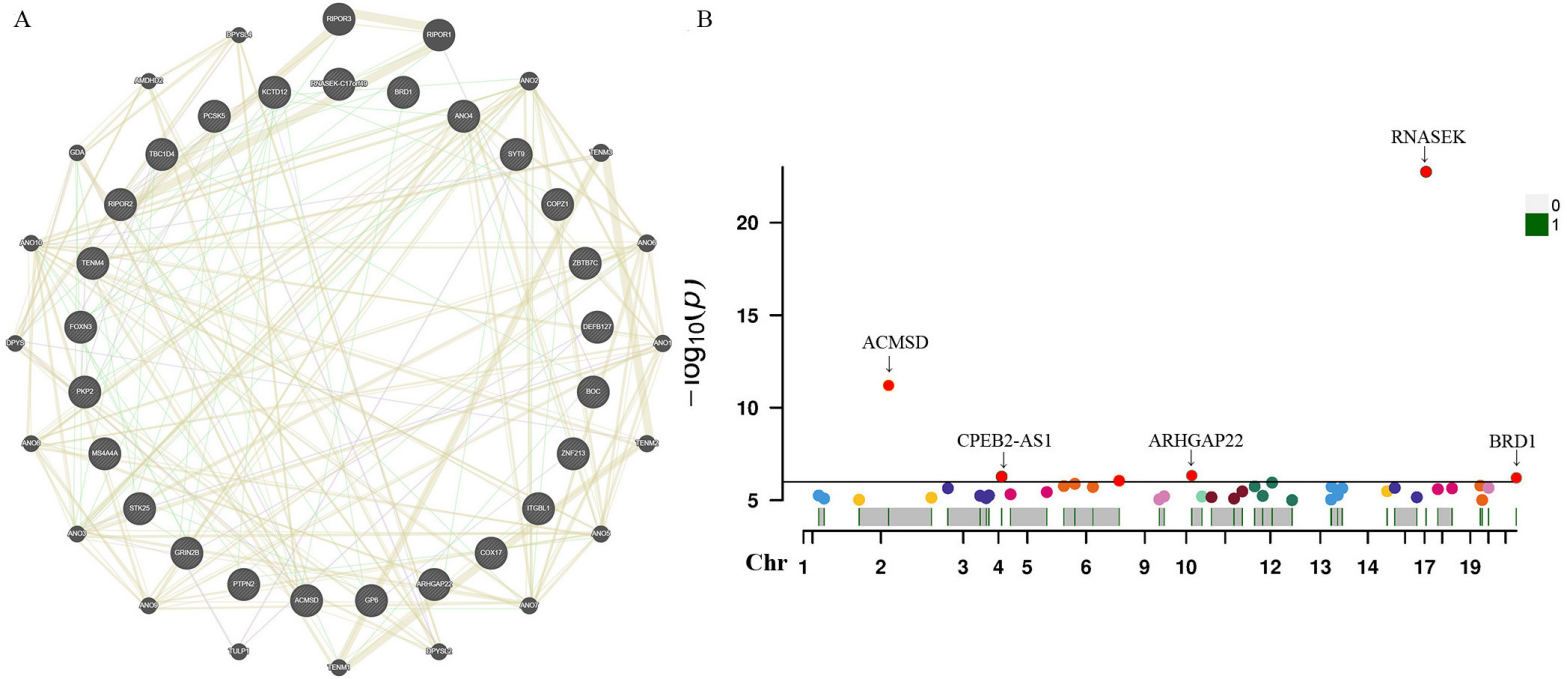
Protein-protein interactive (PPI) network by using GeneMANIA platform **(A)**. Manhattan plot exhibited the traits of genetic instrumental variables associated with three specific metabolites and significant genes were labelled **(B)**. Chr, chromosome.

For these three identified metabolites, we further manually explored metabolism-related SNPs for the second most prevalent traits (alcohol consumption, smoking, blood pressure, BMI, and diabetes). After closely examining the PhenoScannerV2 online tool, we discovered that none of the 41 metabolite-related SNPs were associated with any confounders. The corresponding gene information of instrumental variables unraveling these three metabolites’ associations with the risk of prostate cancer, encompassing effect allele, beta value, SE, R^2^, F-statistics, *P*-value, and closest gene, were demonstrated in **Supplementary Table S5**. Additionally, due to the sample of serum metabolites contained a total of 7824 participants of European ancestry, such a large sample size was sufficient to ensure the robustness and validity of all instrumental variables. Therefore, all the SNPs in this study were biologically plausible. Furthermore, we employed GeneMANIA online tool to construct the protein-protein interactive (PPI) network (**Figure 5A**), and the Manhattan plot exhibited the distribution of genetic locus associated with three specific metabolites and significant genes were labeled in **Figure 5B**. The most significant genes were RNASEK, ACMSD, CPEB2, ARHGAP22, and BRD1.

### 3.6 Single-cell RNA sequencing localization analysis results

**Figure 6:**
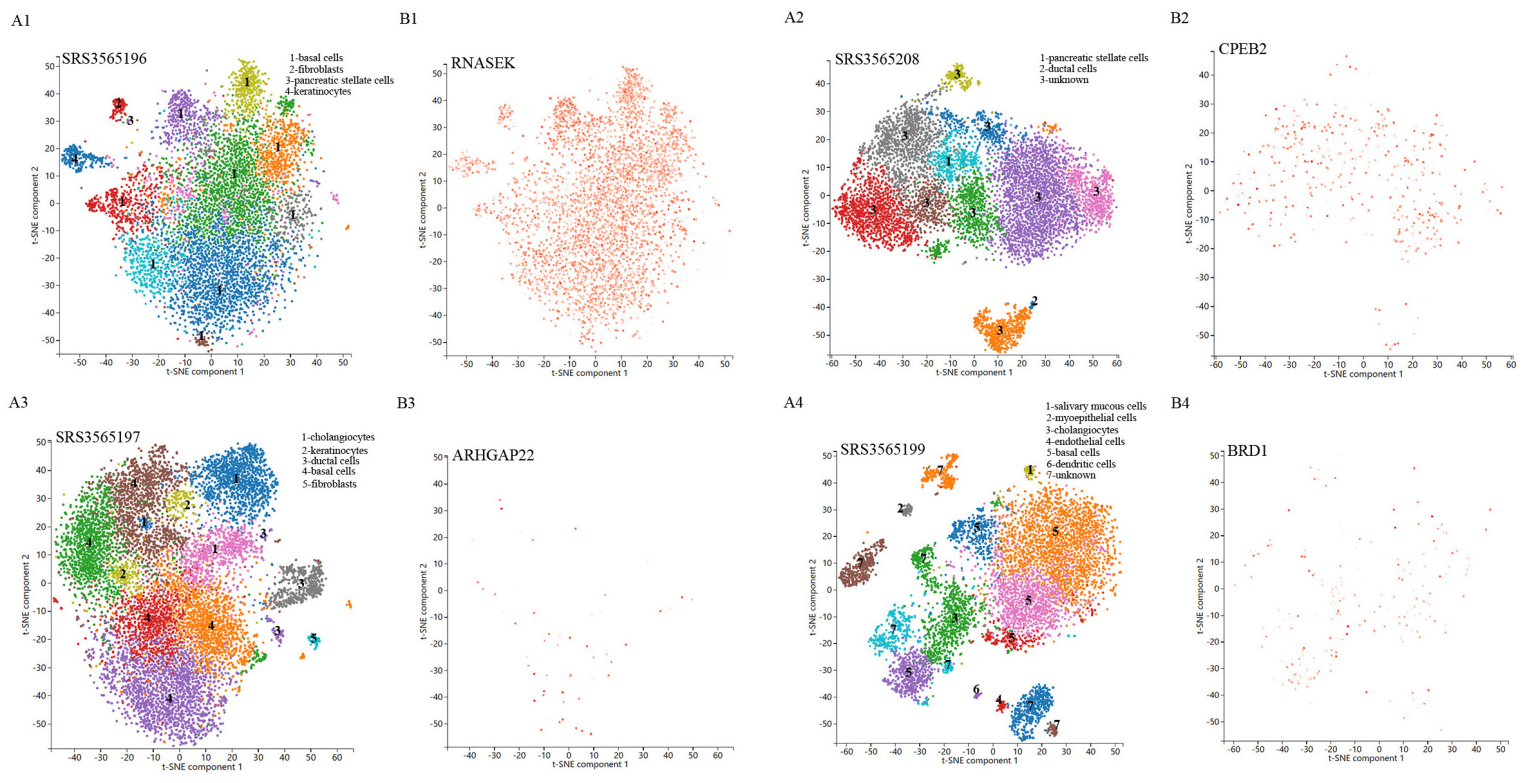
Single-cell RNA sequencing localization analysis of significant genes. **(A1-A4)** Spectral t-SNE plots of all cells analyzed from diverse human prostate tissue samples. **(B1-B4)** The RNASEK, CPEB2, ARHGAP22, and BRD1 expressed levels and distribution in prostate tissue samples.

Single-cell sequencing approach provided cell-specific genetic information and revealed the explicit function and role of target genes. We analyzed scRNA-seq data from four samples, including SRS3565197, SRS3565208, SRS3565199, and SRS3565196 from the Panglao DB database along with cell clustering results and cell type information. After performing rigorous quality control procedures on the data, we employed the t-SNE technique to visualize the high-dimensional scRNA-seq data. As shown in **Figure 6**, data downloaded from Panglao DB demonstrated that RNASEK (**Figure 6A1-B1**), CPEB2 (**Figure 6A2–B2**), ARHGAP22 (**Figure 6A3-B3**), and BRD1 (**Figure 6A4–B4**) were expressed in prostate tissue to varying degrees. Specifically, RNASEK was highly expressed in basal cells, fibroblasts, and keratinocytes. CPEB2 was highly expressed in ductal cells and ARHGAP22 was low expressed in both basal cells and keratinocytes. Moreover, BRD1 was expressed in basal cells and myoepithelial cells to varying degrees. To sum up, prostate tissue cells were segregated into distinct cellular clusters, and RNASEK was designated as the significant gene expression marker. Therefore, we might infer that RNASEK played a significant role in the carcinogenesis of prostate cancer, which aligned with the results of the Manhattan plot exhibition (**Figure 5B**).

### 3.7 Metabolic pathway and enrichment analysis

**Figure 7:**
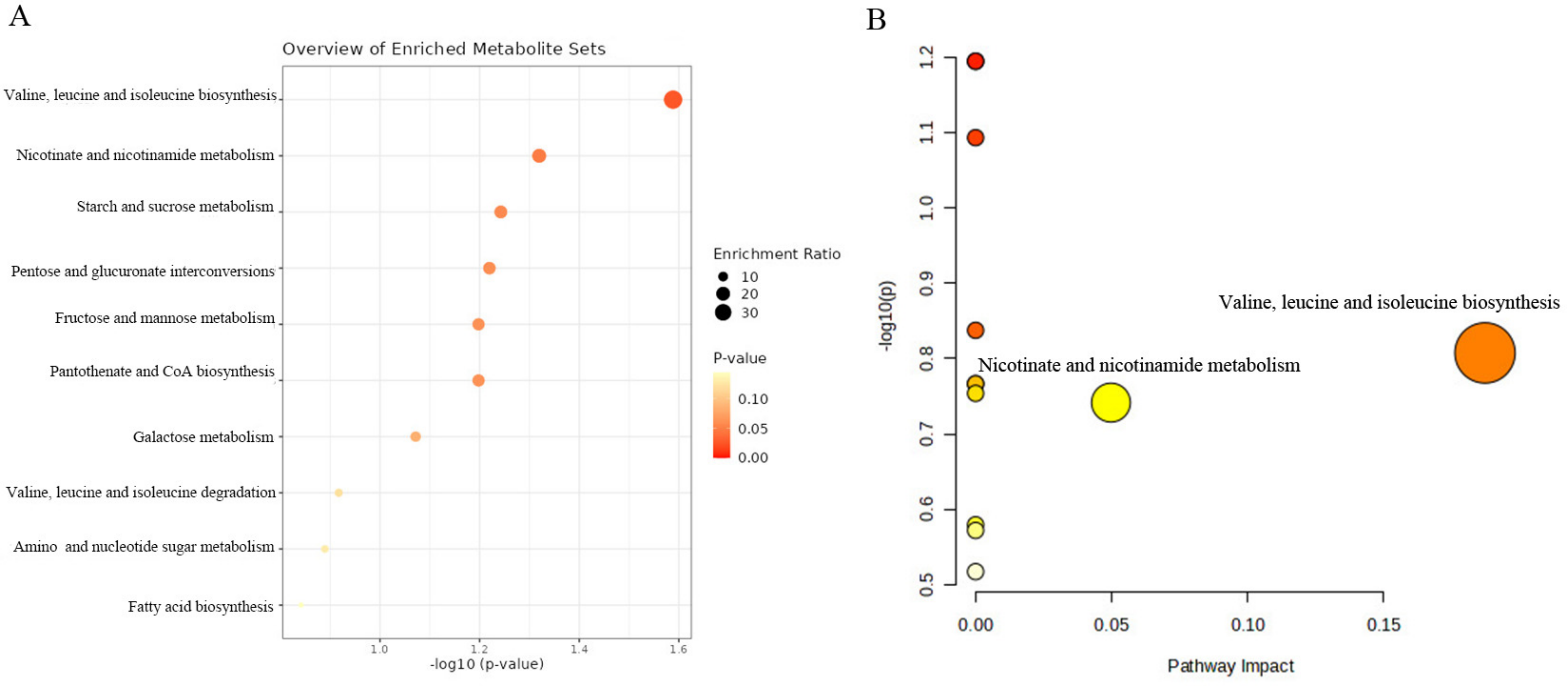
KEGG functional enrichment analysis **(A)** and metabolic pathway analysis **(B)** of identified metabolites by MetaboAnalyst6.0.

We further conducted the metabolic pathway and enrichment analysis utilizing all known metabolites identified through the IVW method (P_IVW_<0.05). The findings of the functional enrichment analysis and the metabolic pathway analysis were exhibited in **Figures 7A, B**. Notably, our study identified two prominent metabolic pathways that played a crucial role in the initiation and progression of prostate cancer (**Supplementary Table S6**) encompassing the valine, leucine and isoleucine biosynthesis pathway (*P*=0.026) and the nicotinate and nicotinamide metabolism pathway (*P*=0.048). The significant metabolite N1-methyl-3-pyridone-4-carboxamide was involved in the nicotinate and nicotinamide metabolism pathway.

## 4 Discussion

In this study, we elucidated the causal associations between 486 genetically determined blood metabolites and the risk of prostate cancer by utilizing genetic variation as IVs in a two-sample Mendelian randomization analysis. Through strict screening criteria and extensive sensitivity analysis, three known metabolites (fructose, N1-methyl-3-pyridone-4-carboxamide, 12-hydroxyeicosatetraenoate (12-HETE)) were identified as the significantly eligible candidates, which were causally linked to the risk of prostate cancer. Additionally, we performed the single-cell RNA sequencing analysis utilizing the Panglao DB database and found significant genes, including RNASEK, CPEB2, ARHGAP22, and BRD1, were enriched in the different cell clusters of prostate tissue. Furthermore, multiple metabolic pathways, especially the valine, leucine and isoleucine biosynthesis pathway and the nicotinate and nicotinamide metabolism pathway, have been found to be involved in the biological process related to the initiation and development of prostate cancer.

Our study demonstrated that genetically determined fructose levels were causally positively associated with the risk of prostate cancer. By coincidence, a previous experimental study conducted by D. V. Carreno et al. showed that dietary fructose promoted prostate cancer growth, which aligns with our findings (37). This research manifested that the expression of fructose transporters was significantly higher in prostate cancer cells compared to benign cells, while there was no significant difference in the expression of glucose transporters (37). Interestingly, they also found that the expression levels of fructose transporters, Glut5 and Glut9, were significantly elevated in clinical specimens of prostate cancer compared to their benign counterparts (37). The serum fructose levels in patients with prostate cancer were significantly elevated compared to those of healthy subjects. Moreover, the consumption of dietary fructose was found to enhance the growth of xenograft tumors derived from prostate cancer cell lines and stimulate the proliferation of prostate cancer cells in patient-derived xenografts. The gene set enrichment analysis of GO and KEGG confirmed that fructose stimulation significantly enriched pathways associated with cell proliferation in prostate cancer cells, thereby promoting their *in vitro* proliferation and invasion (37). Meanwhile, another experimental study conducted by D. Carreno et al. manifested that prostate cancer cells exhibited an enhanced capacity for fructose transportation and metabolism both *in vitro* and *in vivo (* 38). Furthermore, recently a review reported by C. E. Echeverria et al. suggested that the role of fructose as a metabolic substrate in promoting the growth and progression of prostate cancer has been gradually recognized, suggesting that restriction of fructose from the diet could be a useful therapeutic strategy for individuals with prostate cancer (39). To sum up, these studies suggested that fructose promoted prostate cancer cell growth and aggressiveness both in laboratory settings and in living organisms and might represent an alternative energy source for prostate cancer cells, indicating that fructose may be recognized as a crucial metabolic substrate supporting prostate cancer cells and promising therapeutic targets and biomarkers. Nevertheless, another research performed by E. Giovannucci et al. revealed that the elevated consumption of fructose and the increased intake of calcium may potentially lower the likelihood of developing advanced prostate cancer (40). In particular, our findings demonstrated that fructose (OR:1.77, 95%CI:1.05-2.97, P_IVW_=0.031) was the most significant risk factor for prostate cancer. Thus, given the inconsistency and ambiguity of these findings, further studies were essential to detect the precise potential biological mechanism and validate whether fructose could be treated as a promising biomarker in clinical stage stratification and prognosis of prostate cancer.

12-Hydroxyeicosatetraenoic acid (12-HETE) is a metabolite of arachidonic acid (AA) (41). 12-HETE is mainly produced by the release of AA by activated phospholipase A2 (PLA2) and then catalyzed by 12-lipoxygenase (LOX). 12-HETE plays a vital role in various diseases such as cancer, diabetes mellitus, and hypertension, and participates in the occurrence and development of pathological processes such as inflammation and oxidative stress (42, 43). A previous study performed by Q. Liu et al. revealed that the activation of the ILK/NF-kB pathway by 12-HETE was found to participate in the inhibition of cell apoptosis, suggesting a crucial potential mechanism that enhances the survival of ovarian cancer cells (44). Another study conducted by A. Kulkarni et al. demonstrated that 12-lipoxygenases and their eicosanoid metabolites, specifically 12-hydroxyeicosatetraenoate(12-HETE), held significant pathological implications in the context of inflammatory diseases (45). For instance, Nakamura T et al. revealed that 12-HETE promoted late-phase responses in a murine model of allergic rhinitis (46). However, the epidemiological evidence supporting the correlation between 12-HETE and prostate cancer was limited due to its reliance on a case-control study design and constrained by small sample sizes. In this study, we discovered that genetically determined serum 12-HETE level was causally associated with an elevated risk of prostate cancer (OR:1.18, 95%CI: 1.07-1.31, P_IVW_=0.0008), indicating that further investigation may be necessary to validate the findings of observational studies and detect the accurate biological mechanism for 12-HETE on prostate cancer in the future. Finally, the epidemiological evidence of observational studies for the correlation between N1-methyl-3-pyridone-4-carboxamide and prostate cancer was few. In the present study, we observed that a high genetically determined serum level of N1-methyl-3-pyridone-4-carboxamide was found to be causally associated with an elevated risk of prostate cancer (OR:1.29, 95%CI: 1.05-1.58, P_IVW_=0.017). One possible explanation is that, in our study, the sample size of prostate cancer individuals was limited, and underlying confounders were inevitable. Moreover, these results variations may be ascribed to the different choices of IVs and GWAS summary data.

In the present study, the valine, leucine and isoleucine biosynthesis pathway and the nicotinate and nicotinamide metabolism pathway were identified to be associated with the biological process of initiation and progression of prostate cancer by metabolic pathway analysis. Additionally, we discovered that N1-methyl-3-pyridone-4-carboxamide, which participated in the nicotinate and nicotinamide metabolism pathway, may play a crucial role in prostate cancer biological process (metabolism pathway analysis: *P*-value=0.048). These results indicated that N1-methyl-3-pyridone-4-carboxamide may serve as a potential therapeutic target in the occurrence and development of prostate cancer. Hence, further clinical and experimental studies were warranted to elucidate the mechanisms between these identified metabolic pathways and prostate cancer. In the future, it is expected that molecular docking technology will be used in clinical practice to design corresponding drugs for diverse blood metabolites and therapeutic targets. What’s more, our research demonstrated that specific genes, encompassing RNASEK, CPEB2, ARHGAP22, and BRD1, were enriched in the different cell clusters of prostate cancer tissues through the single-cell RNA sequencing localization analysis. Therefore, it is expected to further validate our findings with bulk RNA analysis in the future.

Our study possessed several merits. Firstly, to the best of our knowledge, this is the first MR study to combine metabolomics and single-cell RNA sequencing analysis to systematically evaluate the causal links of human serum metabolites on the risk of prostate cancer. This MR design can mitigate confounding biases often presented in observational studies and provide more strong evidence of causal relationship between exposure and outcome. Secondly, by using the most extensive and current GWAS available as instrumental variables for serum metabolites, we were able to address underlying horizontal pleiotropy and ensure the reliability and robustness of MR results. Thirdly, we identified eligible candidate metabolites causally associated with elevated risk of prostate cancer by multiple criteria and rigorous control process, including pleiotropy, heterogeneity, and LOO analysis. Furthermore, we performed the metabolic pathways analysis for the eligible metabolites and identified the quantitative trait locus of these metabolites from genetic insights.

Nevertheless, the completion of this study required the acknowledgement of multiple limitations. Initially, the selection of IVs was conducted utilizing a widened cutoff (*P*<1e-05), and this might cause consequence bias and false-positive variants. Similarly, a previous study has also utilized the identical threshold when exploring the causal links between 486 serum metabolites and oral cancer (47). Secondly, the individuals of GWAS included in our research were restricted to European ancestry, necessitating further validation in various populations, and larger sample sizes are essential to verify our findings. Thirdly, despite performing diverse sensitivity analyses to validate MR assumptions, we cannot eliminate the impact of horizontal pleiotropy and reverse causality. Fourthly, due to the effects of various serum metabolites on the body being complex and interactive, to fulfill the independent assumption of MR, performing a phenome-wide association study of the instrumental variables may be reasonable. Finally, the precision of our study is intrinsically linked to the size of the sample, underscoring the necessity to enhance the sample for findings validation. Additionally, our MR analysis is limited to public databases and lacks real-world clinical research, indicating that these underlying associations need to be validated through conducting in-depth clinical and experimental studies.

## 5 Conclusions

In conclusion, we conducted a comprehensive MR study to identify three known serum metabolites causally associated with the increased risk of prostate cancer, Additionally, the metabolites causally linked to the risk of prostate cancer were mainly enriched in the valine, leucine and isoleucine biosynthesis pathway and the nicotinate and nicotinamide metabolism pathway. These findings might benefit the comprehension of the biological mechanisms of prostate cancer and facilitate the development of targeted drugs for its treatment.

## Data Availability

The original contributions presented in the study are included in the article/**Supplementary Material**. Further inquiries can be directed to the corresponding authors.
