# Supplementary material for: Causal links of human serum metabolites on the risk of prostate cancer: insights from genome-wide Mendelian randomization, single-cell RNA sequencing, and metabolic pathway analysis
Source: Front Endocrinol (Lausanne). 2024 Nov 12;15:1443330. doi: 10.3389/fendo.2024.1443330 (PMC11590024; doi:10.3389/fendo.2024.1443330)

**Figure S1.** The funnel plots demonstrated the distribution of three identified metabolites instrumental variables (IVs). (A) fructose, (B) N1-methyl-3-pyridone-4-carboxamide, (C) 12-hydroxyeicosatetraenoate (12-HETE).


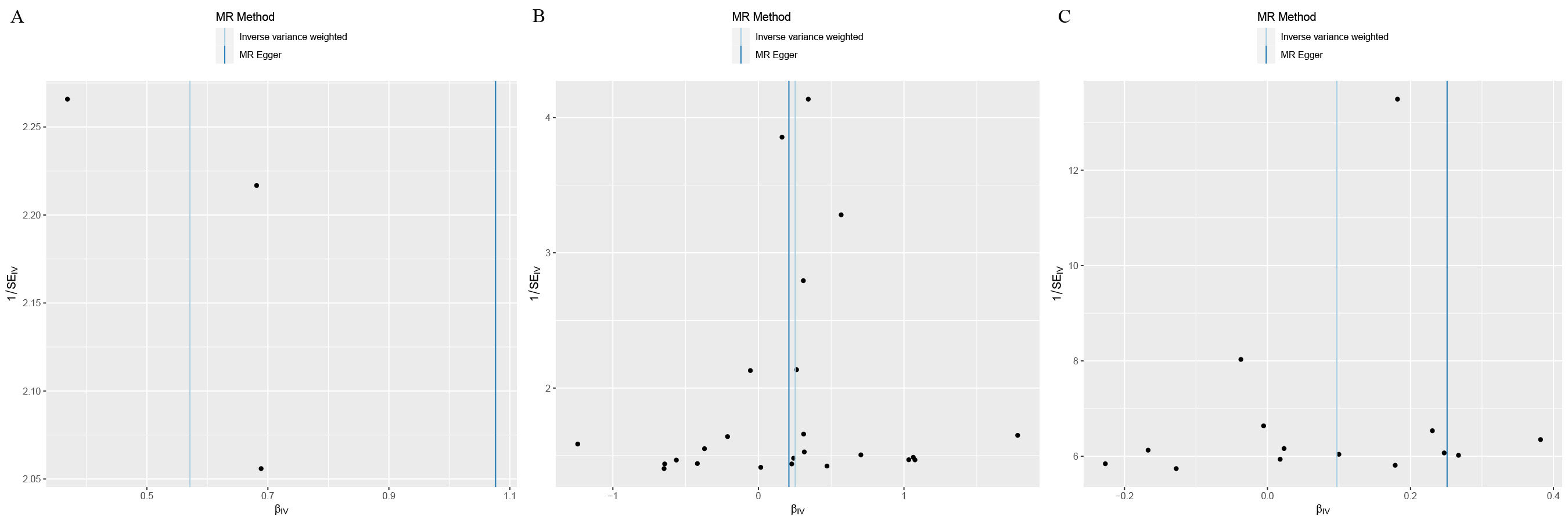

Supplement: Supplementary file 1 [file DataSheet1.zip › Supplementary materials/Supplementary Figure S1.docx]
