# Supplementary material for: Causal links of human serum metabolites on the risk of prostate cancer: insights from genome-wide Mendelian randomization, single-cell RNA sequencing, and metabolic pathway analysis
Source: Front Endocrinol (Lausanne). 2024 Nov 12;15:1443330. doi: 10.3389/fendo.2024.1443330 (PMC11590024; doi:10.3389/fendo.2024.1443330)
Supplement: Supplementary file 1 [file DataSheet1.zip › Supplementary materials/Supplementary Table S1.docx]

**Table S1.** Brief characteristics description of 486 human serum metabolites and prostate cancer GWAS cohorts involved in this study.

| **Exposure or outcome** | **Source** | **Sample size** | **Ancestry** | **Access Link** | **PMID** |
| --- | --- | --- | --- | --- | --- |
| 486 human serum metabolites | KORA F4 and UK Twin study | 7,824 participants | European | http://www.ncbi.nlm.nih.gov/pubmed/24816252 | 24816252 |
| Prostate cancer | PRACTICAL Consortium | 79,148 cases and 61,106 controls | European | http://gwas.mrcieu.ac.uk/datasets/ieu-b-85/ | 29892016 |
