# Supplementary material for: Causal links of human serum metabolites on the risk of prostate cancer: insights from genome-wide Mendelian randomization, single-cell RNA sequencing, and metabolic pathway analysis
Source: Front Endocrinol (Lausanne). 2024 Nov 12;15:1443330. doi: 10.3389/fendo.2024.1443330 (PMC11590024; doi:10.3389/fendo.2024.1443330)
Supplement: Supplementary file 1 [file DataSheet1.zip › Supplementary materials/Supplementary Table S3.docx]

**Table S3.** MR analysis of the associations between 30 eligible candidate metabolites and prostate cancer.

| **Candidate human serum metabolites** | | **Number of SNPs** | **OR (95% CI)** | **P-value** |
| --- | --- | --- | --- | --- |
| **Arabinose** | |  |  |  |
|  | Inverse variance weighted | 5 | 0.84 (0.74- 0.96) | 0.011 |
|  | Weighted median | 5 | 0.85 (0.71 - 1.02) | 0.082 |
|  | MR Egger | 5 | 0.89 (0.70- 1.12) | 0.400 |
|  | Simple mode | 5 | 0.77 (0.58 - 1.04) | 0.166 |
|  | Weighted mode | 5 | 0.85 (0.72- 1.01) | 0.139 |
| **Fructose** | |  |  |  |
|  | Inverse variance weighted | 3 | 1.77 (1.05 -2.97) | 0.031 |
|  | Weighted median | 3 | 1.90(1.00- 3.62) | 0.049 |
|  | MR Egger | 3 | 1.39 (0.51- 4.28) | 0.634 |
|  | Simple mode | 3 | 1.98 (0.97- 4.07) | 0.202 |
|  | Weighted mode | 3 | 1.98 (0.93- 4.21) | 0.217 |
| **Deoxycholate** | |  |  |  |
|  | Inverse variance weighted | 17 | 1.20 (1.04 - 1.39) | 0.013 |
|  | Weighted median | 17 | 1.23 (1.05 - 1.44) | 0.012 |
|  | MR Egger | 17 | 1.38 (0.99 - 1.92) | 0.081 |
|  | Simple mode | 17 | 1.25 (0.94 - 1.65) | 0.140 |
|  | Weighted mode | 17 | 1.26 (1.02 - 1.56) | 0.049 |
| **Salicylate** | |  |  |  |
|  | Inverse variance weighted | 18 | 1.04 (1.01 - 1.07) | 0.012 |
|  | Weighted median | 18 | 1.02 (0.99 - 1.06) | 0.163 |
|  | MR Egger | 18 | 1.03 (0.99 - 1.07) | 0.228 |
|  | Simple mode | 18 | 1.05 (0.99 - 1.11) | 0.146 |
|  | Weighted mode | 18 | 1.03 (1.00 - 1.06) | 0.115 |
| **Valine** | |  |  |  |
|  | Inverse variance weighted | 6 | 2.02 (1.04 -3.92) | 0.037 |
|  | Weighted median | 6 | 1.76 (0.79- 3.91) | 0.166 |
|  | MR Egger | 6 | 1.51 (0.62 - 3.70) | 0.415 |
|  | Simple mode | 6 | 2.01 (0.65 - 6.24) | 0.282 |
|  | Weighted mode | 6 | 1.69 (0.74 - 3.90) | 0.270 |
| **Phosphate** | |  |  |  |
|  | Inverse variance weighted | 5 | 2.00 (1.01 -3.96) | 0.047 |
|  | Weighted median | 5 | 2.11 (0.90 -4.99) | 0.088 |
|  | MR Egger | 5 | 3.58 (1.22 - 10.45) | 0.102 |
|  | Simple mode | 5 | 2.06 (0.66 - 6.43) | 0.279 |
|  | Weighted mode | 5 | 2.26 (0.94 - 5.41) | 0.143 |
| **X-03003** | |  |  |  |
|  | Inverse variance weighted | 9 | 5.25 (1.09 - 25.37) | 0.039 |
|  | Weighted median | 9 | 2.99 (1.45 - 6.16) | 0.003 |
|  | MR Egger | 9 | 0.00 (0.00- 20.96) | 0.217 |
|  | Simple mode | 9 | 3.42 (1.11 - 10.53) | 0.065 |
|  | Weighted mode | 9 | 3.50 (1.22 - 10.00) | 0.048 |
| **Benzoate** | |  |  |  |
|  | Inverse variance weighted | 42 | 0.73 (0.54 -1.00) | 0.049 |
|  | Weighted median | 42 | 0.72 (0.46 -1.11) | 0.134 |
|  | MR Egger | 42 | 0.70 (0.27 - 1.85) | 0.478 |
|  | Simple mode | 42 | 0.70 (0.30- 1.65) | 0.424 |
|  | Weighted mode | 42 | 0.69 (0.35 - 1.38) | 0.302 |
| **2-hydroxyisobutyrate** | |  |  |  |
|  | Inverse variance weighted | 18 | 0.74 (0.55- 1.00) | 0.047 |
|  | Weighted median | 18 | 0.87 (0.56 - 1.34) | 0.527 |
|  | MR Egger | 18 | 0.78 (0.33 - 1.81) | 0.567 |
|  | Simple mode | 18 | 0.92 (0.48 - 1.74) | 0.798 |
|  | Weighted mode | 18 | 0.90 (0.51- 1.57) | 0.713 |
| **4-methyl-2-oxopentanoate** | |  |  |  |
|  | Inverse variance weighted | 14 | 1.54 (1.07 -2.21) | 0.019 |
|  | Weighted median | 14 | 1.57 (0.96- 2.57) | 0.073 |
|  | MR Egger | 14 | 1.16 (0.33 - 4.16) | 0.818 |
|  | Simple mode | 14 | 1.52 (0.73- 3.17) | 0.279 |
|  | Weighted mode | 14 | 1.45 (0.73- 2.87) | 0.306 |
| **X-10395** | |  |  |  |
|  | Inverse variance weighted | 29 | 0.82 (0.68 - 1.00) | 0.046 |
|  | Weighted median | 29 | 0.85 (0.65- 1.10) | 0.223 |
|  | MR Egger | 29 | 0.79 (0.61 - 1.03) | 0.089 |
|  | Simple mode | 29 | 1.15 (0.67 - 1.95) | 0.620 |
|  | Weighted mode | 29 | 0.82 (0.67- 1.00) | 0.065 |
| **X-10810** | |  |  |  |
|  | Inverse variance weighted | 16 | 0.82 (0.68 -0.99) | 0.040 |
|  | Weighted median | 16 | 0.76 (0.58 - 0.99) | 0.042 |
|  | MR Egger | 16 | 0.71 (0.54- 0.93) | 0.025 |
|  | Simple mode | 16 | 0.95 (0.53 - 1.71) | 0.865 |
|  | Weighted mode | 16 | 0.76 (0.59- 0.97) | 0.044 |
| **DSGEGDFXAEGGGVR** | |  |  |  |
|  | Inverse variance weighted | 13 | 0.85 (0.74 - 0.98) | 0.028 |
|  | Weighted median | 13 | 0.84 (0.69 - 1.03) | 0.089 |
|  | MR Egger | 13 | 0.74 (0.48- 1.15) | 0.212 |
|  | Simple mode | 13 | 0.71 (0.49- 1.03) | 0.097 |
|  | Weighted mode | 13 | 0.72 (0.51 - 1.02) | 0.087 |
| **Caprylate (8:0)** | |  |  |  |
|  | Inverse variance weighted | 44 | 1.49 (1.07 -2.07) | 0.019 |
|  | Weighted median | 44 | 1.25 (0.83 - 1.86) | 0.283 |
|  | MR Egger | 44 | 1.42 (0.66 - 3.05) | 0.371 |
|  | Simple mode | 44 | 1.99 (1.00- 3.94) | 0.055 |
|  | Weighted mode | 44 | 1.19 (0.70- 2.00) | 0.525 |
| **X-07765** | |  |  |  |
|  | Inverse variance weighted | 13 | 0.88 (0.81- 0.96) | 0.002 |
|  | Weighted median | 13 | 0.84 (0.75- 0.95) | 0.004 |
|  | MR Egger | 13 | 0.94 (0.81- 1.08) | 0.409 |
|  | Simple mode | 13 | 0.81 (0.68- 0.98) | 0.053 |
|  | Weighted mode | 13 | 0.83 (0.71- 0.97) | 0.035 |
| **X-11315** | |  |  |  |
|  | Inverse variance weighted | 28 | 1.20 (1.02 - 1.42) | 0.030 |
|  | Weighted median | 28 | 1.18 (0.94 - 1.50) | 0.155 |
|  | MR Egger | 28 | 1.07 (0.80 - 1.43) | 0.669 |
|  | Simple mode | 28 | 1.02 (0.68 - 1.51) | 0.939 |
|  | Weighted mode | 28 | 1.11 (0.83 - 1.48) | 0.483 |
| **X-11438** | |  |  |  |
|  | Inverse variance weighted | 23 | 0.82 (0.67 - 1.00) | 0.048 |
|  | Weighted median | 23 | 0.88 (0.70 - 1.10) | 0.257 |
|  | MR Egger | 23 | 0.71 (0.48 - 1.06) | 0.111 |
|  | Simple mode | 23 | 0.81 (0.54 -1.21) | 0.317 |
|  | Weighted mode | 23 | 0.86 (0.64- 1.17) | 0.355 |
| **X-11537** | |  |  |  |
|  | Inverse variance weighted | 6 | 1.33 (1.06 -1.67) | 0.013 |
|  | Weighted median | 6 | 1.25 (0.96 - 1.63) | 0.102 |
|  | MR Egger | 6 | 1.15 (0.36 - 3.71) | 0.829 |
|  | Simple mode | 6 | 1.23 (0.84 - 1.80) | 0.347 |
|  | Weighted mode | 6 | 1.24 (0.87 - 1.76) | 0.296 |
| **X-12063** | |  |  |  |
|  | Inverse variance weighted | 16 | 1.12 (1.02 - 1.23) | 0.023 |
|  | Weighted median | 16 | 1.09 (0.96- 1.23) | 0.175 |
|  | MR Egger | 16 | 1.06 (0.92 - 1.22) | 0.453 |
|  | Simple mode | 16 | 1.10 (0.89 - 1.37) | 0.389 |
|  | Weighted mode | 16 | 1.07 (0.97 - 1.19) | 0.213 |
| **N1-methyl-3-pyridone-4-carboxamide** | |  |  |  |
|  | Inverse variance weighted | 24 | 1.29 (1.05- 1.58) | 0.017 |
|  | Weighted median | 24 | 1.35 (1.01- 1.79) | 0.043 |
|  | MR Egger | 24 | 1.23 (0.83- 1.82) | 0.304 |
|  | Simple mode | 24 | 1.30 (0.81- 2.11) | 0.292 |
|  | Weighted mode | 24 | 1.33 (0.97 - 1.82) | 0.093 |
| **12-hydroxyeicosatetraenoate (12-HETE)** | |  |  |  |
|  | Inverse variance weighted | 14 | 1.18 (1.07 - 1.31) | 0.0008 |
|  | Weighted median | 14 | 1.10 (1.01 - 1.20) | 0.027 |
|  | MR Egger | 14 | 1.29 (1.00 - 1.66) | 0.076 |
|  | Simple mode | 14 | 1.19 (0.97- 1.47) | 0.127 |
|  | Weighted mode | 14 | 1.19 (1.03 - 1.38) | 0.037 |
|  | **X-12717** |  |  |  |
|  | Inverse variance weighted | 9 | 0.91 (0.82 - 1.00) | 0.049 |
|  | Weighted median | 9 | 0.96 (0.84 - 1.09) | 0.501 |
|  | MR Egger | 9 | 1.08 (0.70 - 1.65) | 0.741 |
|  | Simple mode | 9 | 0.96 (0.79- 1.17) | 0.693 |
|  | Weighted mode | 9 | 0.97 (0.81 - 1.16) | 0.732 |
|  | **X-12726** |  |  |  |
|  | Inverse variance weighted | 21 | 0.89 (0.80 - 0.99) | 0.028 |
|  | Weighted median | 21 | 0.88 (0.76 - 1.01) | 0.063 |
|  | MR Egger | 21 | 0.85 (0.74 - 0.96) | 0.019 |
|  | Simple mode | 21 | 0.97 (0.75- 1.25) | 0.802 |
|  | Weighted mode | 21 | 0.88 (0.79 - 0.98) | 0.035 |
|  | **Stachydrine** |  |  |  |
|  | Inverse variance weighted | 6 | 0.90 (0.80 - 1.00) | 0.049 |
|  | Weighted median | 6 | 0.94 (0.82 - 1.08) | 0.393 |
|  | MR Egger | 6 | 1.03 (0.68 - 1.55) | 0.898 |
|  | Simple mode | 6 | 0.95 (0.78- 1.15) | 0.604 |
|  | Weighted mode | 6 | 0.95 (0.78 - 1.15) | 0.613 |
|  | **X-12851** |  |  |  |
|  | Inverse variance weighted | 8 | 1.07 (1.02 - 1.13) | 0.004 |
|  | Weighted median | 8 | 1.07 (1.02 - 1.13) | 0.013 |
|  | MR Egger | 8 | 1.09 (1.02 - 1.16) | 0.037 |
|  | Simple mode | 8 | 1.03 (0.88- 1.20) | 0.732 |
|  | Weighted mode | 8 | 1.08 (1.03 - 1.13) | 0.017 |
|  | **Hydroxyisovaleroyl carnitine** |  |  |  |
|  | Inverse variance weighted | 8 | 0.77 (0.60 - 0.99) | 0.045 |
|  | Weighted median | 8 | 0.84 (0.60 - 1.18) | 0.313 |
|  | MR Egger | 8 | 0.98 (0.61 - 1.57) | 0.921 |
|  | Simple mode | 8 | 0.87 (0.56- 1.34) | 0.545 |
|  | Weighted mode | 8 | 0.86 (0.59 - 1.27) | 0.481 |
|  | **1-myristoylglycerophosphocholine** |  |  |  |
|  | Inverse variance weighted | 6 | 1.48 (1.10 - 1.99) | 0.009 |
|  | Weighted median | 6 | 1.32 (0.89 - 1.94) | 0.168 |
|  | MR Egger | 6 | 1.06 (0.52 - 2.18) | 0.879 |
|  | Simple mode | 6 | 1.30 (0.78- 2.17) | 0.359 |
|  | Weighted mode | 6 | 1.31 (0.90 - 1.92) | 0.215 |
|  | **X-14086** |  |  |  |
|  | Inverse variance weighted | 14 | 0.73 (0.60 - 0.90) | 0.003 |
|  | Weighted median | 14 | 0.74 (0.59 - 0.93) | 0.009 |
|  | MR Egger | 14 | 1.03 (0.54 - 1.96) | 0.927 |
|  | Simple mode | 14 | 0.74 (0.51- 1.08) | 0.142 |
|  | Weighted mode | 14 | 0.72 (0.49 - 1.05) | 0.111 |
|  | **X-14473** |  |  |  |
|  | Inverse variance weighted | 11 | 1.36 (1.13 - 1.64) | 0.001 |
|  | Weighted median | 11 | 1.33 (1.03 - 1.71) | 0.031 |
|  | MR Egger | 11 | 1.60 (0.77 - 3.32) | 0.239 |
|  | Simple mode | 11 | 1.51 (1.00- 2.29) | 0.077 |
|  | Weighted mode | 11 | 1.30 (0.91 - 1.86) | 0.174 |
|  | **X-14632** |  |  |  |
|  | Inverse variance weighted | 18 | 0.87 (0.77 - 0.99) | 0.034 |
|  | Weighted median | 18 | 0.91 (0.78 - 1.07) | 0.274 |
|  | MR Egger | 18 | 1.04 (0.88 - 1.23) | 0.678 |
|  | Simple mode | 18 | 0.71 (0.48- 1.04) | 0.098 |
|  | Weighted mode | 18 | 0.93 (0.79 - 1.10) | 0.399 |

**Abbreviations:** SNP, single nucleotide polymorphism; OR, odds ratio; CI, confidence interval.
