# Supplementary material for: Causal links of human serum metabolites on the risk of prostate cancer: insights from genome-wide Mendelian randomization, single-cell RNA sequencing, and metabolic pathway analysis
Source: Front Endocrinol (Lausanne). 2024 Nov 12;15:1443330. doi: 10.3389/fendo.2024.1443330 (PMC11590024; doi:10.3389/fendo.2024.1443330)
Supplement: Supplementary file 1 [file DataSheet1.zip › Supplementary materials/Supplementary Table S4.docx]

**Table S4.** Sensitivity analysis results for the 30 metabolites identified by the IVW method.

| **Metabolites** | **Subcategory** | **Pleiotropy** | | **Heterogeneity** | | | |
| --- | --- | --- | --- | --- | --- | --- | --- |
|  |  | **MR-Egger Intercept test** | | **MR-Egger** | | **IVW** | |
|  |  | Intercept | *P-*value | Q-statistic | *P-*value | Q-statistic | *P-*value |
| Arabinose | Carbohydrate | 0.0007 | 0.929 | 9.590 | 0.143 | 9.604 | 0.212 |
| Fructose | Carbohydrate | -0.0127 | 0.669 | 0.0002 | 0.989 | 0.329 | 0.849 |
| Deoxycholate | Lipid | -0.0074 | 0.394 | 27.334 | 0.026 | 28.741 | 0.026 |
| Salicylate | Xenobiotics | 0.0056 | 0.361 | 28.881 | 0.025 | 30.478 | 0.023 |
| Valine | Amino acid | 0.0055 | 0.399 | 1.663 | 0.797 | 2.552 | 0.769 |
| Phosphate | Energy | -0.0099 | 0.276 | 2.745 | 0.433 | 4.512 | 0.341 |
| X-03003 | Unknown | 0.1140 | 0.132 | 55.775 | 1.05E-09 | 78.873 | 8.24E-14 |
| Benzoate | Xenobiotics | 0.0005 | 0.928 | 46.215 | 0.231 | 46.224 | 0.265 |
| 2-hydroxyisobutyrate | Amino acid | -0.0008 | 0.905 | 16.737 | 0.403 | 16.753 | 0.471 |
| 4-methyl-2-oxopentanoate | Amino acid | 0.0044 | 0.662 | 7.570 | 0.818 | 7.772 | 0.858 |
| X-10395 | Unknown | 0.0012 | 0.663 | 34.819 | 0.144 | 35.070 | 0.168 |
| X-10810 | Unknown | 0.0061 | 0.158 | 12.878 | 0.536 | 15.104 | 0.444 |
| DSGEGDFXAEGGGVR | Peptide | 0.0059 | 0.533 | 8.646 | 0.655 | 9.061 | 0.698 |
| Caprylate (8:0) | Lipid | 0.0006 | 0.897 | 76.767 | 8.00E-04 | 76.798 | 1.20E-03 |
| X-07765 | Unknown | -0.0059 | 0.308 | 10.722 | 0.467 | 11.865 | 0.457 |
| X-11315 | Unknown | 0.0034 | 0.339 | 21.096 | 0.737 | 22.047 | 0.735 |
| X-11438 | Unknown | 0.0046 | 0.435 | 35.018 | 0.028 | 36.075 | 0.029 |
| X-11537 | Unknown | 0.0057 | 0.813 | 5.889 | 0.208 | 5.983 | 0.308 |
| X-12063 | Unknown | 0.0043 | 0.316 | 17.048 | 0.254 | 18.365 | 0.244 |
| N1-methyl-3-pyridone-4-carboxamide | Nucleotide | 0.0011 | 0.798 | 25.769 | 0.262 | 25.847 | 0.308 |
| 12-hydroxyeicosatetraenoate (12-HETE) | Lipid | -0.0114 | 0.232 | 15.332 | 0.224 | 17.361 | 0.183 |
| X-12717 | Unknown | -0.0136 | 0.437 | 7.003 | 0.429 | 7.682 | 0.465 |
| X-12726 | Unknown | 0.0041 | 0.222 | 21.107 | 0.331 | 22.880 | 0.295 |
| Stachydrine | Xenobiotics | -0.0110 | 0.532 | 1.963 | 0.742 | 2.429 | 0.787 |
| X-12851 | Unknown | -0.0056 | 0.454 | 14.143 | 0.028 | 15.651 | 0.029 |
| Hydroxyisovaleroyl carnitine | Amino acid | -0.0090 | 0.299 | 3.066 | 0.801 | 4.357 | 0.738 |
| 1-myristoylglycerophosphocholine | Lipid | 0.0099 | 0.375 | 4.596 | 0.331 | 5.737 | 0.333 |
| X-14086 | Unknown | -0.0134 | 0.298 | 19.326 | 0.081 | 21.232 | 0.069 |
| X-14473 | Unknown | -0.0050 | 0.662 | 6.732 | 0.665 | 6.937 | 0.731 |
| X-14632 | Unknown | -0.0121 | 0.020 | 17.931 | 0.328 | 25.396 | 0.086 |
