# Supplementary material for: Causal links of human serum metabolites on the risk of prostate cancer: insights from genome-wide Mendelian randomization, single-cell RNA sequencing, and metabolic pathway analysis
Source: Front Endocrinol (Lausanne). 2024 Nov 12;15:1443330. doi: 10.3389/fendo.2024.1443330 (PMC11590024; doi:10.3389/fendo.2024.1443330)
Supplement: Supplementary file 1 [file DataSheet1.zip › Supplementary materials/Supplementary Table S5.docx]

**Table S5.** SNPs detailed information of 3 identified metabolites in this MR study.

| Serum metabolites | SNP | | Chr | Position | Effect allele | Beta | SE | R^2^ | F | *P*-value | Closest  gene |
| --- | --- | --- | --- | --- | --- | --- | --- | --- | --- | --- | --- |
| Fructose | | rs2456584 | 19 | 51313145 | A | -0.0179 | 0.0037 | 0.003 | 23.39 | 1.64E-06 | C19orf48 |
| Fructose | | rs4663119 | 2 | 235172988 | A | 0.0331 | 0.0074 | 0.002 | 20.00 | 7.32E-06 | STK25 |
| Fructose | | rs661454 | 6 | 50620678 | A | 0.0317 | 0.0065 | 0.003 | 23.78 | 1.31E-06 | RP11-228O6.2 |
| N1-methyl-3-pyridone-4-carboxamide | | rs11230225 | 11 | 60038176 | T | 0.0366 | 0.0082 | 0.011 | 19.89 | 8.16E-06 | MS4A4A |
| N1-methyl-3-pyridone-4-carboxamide | | rs12429935 | 13 | 91507351 | T | -0.0586 | 0.0129 | 0.012 | 20.61 | 5.31E-06 | LINC00410 |
| N1-methyl-3-pyridone-4-carboxamide | | rs12795652 | 11 | 79185332 | A | -0.0203 | 0.0044 | 0.003 | 21.28 | 3.34E-06 | TENM4 |
| N1-methyl-3-pyridone-4-carboxamide | | rs12953402 | 18 | 45852017 | T | 0.0165 | 0.0035 | 0.003 | 22.22 | 2.29E-06 | ZBTB7C |
| N1-methyl-3-pyridone-4-carboxamide | | rs13021657 | 2 | 66829249 | T | -0.0519 | 0.0117 | 0.003 | 19.67 | 9.43E-06 | LINC01798 |
| N1-methyl-3-pyridone-4-carboxamide | | rs1421106 | 12 | 14125734 | T | -0.0236 | 0.0049 | 0.003 | 23.19 | 1.79E-06 | GRIN2B |
| N1-methyl-3-pyridone-4-carboxamide | | rs1674726 | 6 | 153858614 | A | -0.0137 | 0.0028 | 0.003 | 23.93 | 8.82E-07 | RP11-15G8.1 |
| N1-methyl-3-pyridone-4-carboxamide | | rs16830291 | 3 | 119385140 | T | -0.0121 | 0.0027 | 0.003 | 20.08 | 5.55E-06 | COX17 |
| N1-methyl-3-pyridone-4-carboxamide | | rs16939910 | 18 | 12837993 | A | -0.0292 | 0.0062 | 0.013 | 22.16 | 2.52E-06 | PTPN2 |
| N1-methyl-3-pyridone-4-carboxamide | | rs17754631 | 3 | 23806758 | A | -0.0161 | 0.0034 | 0.003 | 22.42 | 2.25E-06 | AC020626.1 |
| N1-methyl-3-pyridone-4-carboxamide | | rs2328991 | 13 | 77397101 | C | 0.0204 | 0.0043 | 0.003 | 22.50 | 1.88E-06 | KCTD12 |
| N1-methyl-3-pyridone-4-carboxamide | | rs248670 | 5 | 6055696 | T | 0.0122 | 0.0027 | 0.003 | 20.41 | 4.78E-06 | LINC02142 |
| N1-methyl-3-pyridone-4-carboxamide | | rs303031 | 6 | 25036980 | A | -0.0125 | 0.0026 | 0.003 | 23.11 | 1.72E-06 | FAM65B |
| N1-methyl-3-pyridone-4-carboxamide | | rs3783853 | 14 | 89803269 | A | 0.0124 | 0.0027 | 0.003 | 21.09 | 3.27E-06 | FOXN3 |
| N1-methyl-3-pyridone-4-carboxamide | | rs4073619 | 11 | 7490548 | T | -0.0119 | 0.0026 | 0.003 | 20.94 | 6.83E-06 | SYT9 |
| N1-methyl-3-pyridone-4-carboxamide | | rs4878060 | 9 | 89955458 | T | -0.0123 | 0.0027 | 0.003 | 20.74 | 6.17E-06 | SNORA26 |
| N1-methyl-3-pyridone-4-carboxamide | | rs6055874 | 20 | 144382 | T | 0.0125 | 0.0026 | 0.003 | 23.11 | 2.15E-06 | DEFB127 |
| N1-methyl-3-pyridone-4-carboxamide | | rs6430553 | 2 | 135631400 | T | -0.0181 | 0.0026 | 0.007 | 48.45 | 6.29E-12 | ACMSD |
| N1-methyl-3-pyridone-4-carboxamide | | rs7133996 | 12 | 32931925 | T | -0.0151 | 0.0033 | 0.003 | 20.93 | 5.83E-06 | PKP2 |
| N1-methyl-3-pyridone-4-carboxamide | | rs7327971 | 13 | 102304708 | A | -0.0125 | 0.0026 | 0.003 | 23.11 | 2.25E-06 | ITGBL1 |
| N1-methyl-3-pyridone-4-carboxamide | | rs733017 | 16 | 54517482 | T | -0.0118 | 0.0026 | 0.003 | 20.59 | 6.94E-06 | LINC02183 |
| N1-methyl-3-pyridone-4-carboxamide | | rs935270 | 10 | 49757251 | A | -0.0135 | 0.0027 | 0.003 | 24.99 | 4.66E-07 | ARHGAP22 |
| N1-methyl-3-pyridone-4-carboxamide | | rs9451877 | 6 | 92701566 | A | -0.0943 | 0.0198 | 0.013 | 22.66 | 1.92E-06 | RP11-374I15.1 |
| N1-methyl-3-pyridone-4-carboxamide | | rs9825012 | 3 | 112951832 | T | 0.0434 | 0.0097 | 0.012 | 19.99 | 7.70E-06 | BOC |
| 12-hydroxyeicosatetraenoate (12-HETE) | | rs1053871 | 16 | 3181963 | T | 0.0527 | 0.0111 | 0.008 | 22.52 | 2.14E-06 | ZNF213 |
| 12-hydroxyeicosatetraenoate (12-HETE) | | rs10869731 | 9 | 78811377 | T | -0.0647 | 0.0146 | 0.007 | 19.62 | 9.01E-06 | PCSK5 |
| 12-hydroxyeicosatetraenoate (12-HETE) | | rs11110563 | 12 | 101256840 | T | -0.0485 | 0.011 | 0.007 | 19.43 | 9.88E-06 | ANO4 |
| 12-hydroxyeicosatetraenoate (12-HETE) | | rs11163633 | 1 | 83477356 | A | 0.0508 | 0.0112 | 0.007 | 20.56 | 5.60E-06 | LINC01362 |
| 12-hydroxyeicosatetraenoate (12-HETE) | | rs12263193 | 10 | 73641906 | A | -0.0584 | 0.0129 | 0.007 | 20.48 | 6.34E-06 | RP11-472G21.2 |
| 12-hydroxyeicosatetraenoate (12-HETE) | | rs12429941 | 13 | 76031644 | A | -0.1462 | 0.033 | 0.007 | 19.61 | 9.29E-06 | TBC1D4 |
| 12-hydroxyeicosatetraenoate (12-HETE) | | rs138832 | 22 | 50178082 | A | 0.0779 | 0.0156 | 0.014 | 24.91 | 6.18E-07 | BRD1 |
| 12-hydroxyeicosatetraenoate (12-HETE) | | rs1397793 | 5 | 90471451 | A | -0.0564 | 0.0122 | 0.008 | 21.36 | 3.65E-06 | CTD-2151A2.3 |
| 12-hydroxyeicosatetraenoate (12-HETE) | | rs1494950 | 4 | 14967169 | A | 0.1843 | 0.0367 | 0.009 | 25.19 | 5.31E-07 | CPEB2-AS1 |
| 12-hydroxyeicosatetraenoate (12-HETE) | | rs1671152 | 19 | 55526345 | T | -0.0666 | 0.0151 | 0.007 | 19.44 | 9.75E-06 | GP6 |
| 12-hydroxyeicosatetraenoate (12-HETE) | | rs2271316 | 17 | 6915401 | C | 0.1133 | 0.0113 | 0.036 | 100.46 | 1.79E-23 | RNASEK-C17orf49 |
| 12-hydroxyeicosatetraenoate (12-HETE) | | rs6580981 | 12 | 54723028 | A | -0.0551 | 0.0113 | 0.009 | 23.76 | 1.12E-06 | COPZ1 |
| 12-hydroxyeicosatetraenoate (12-HETE) | | rs684448 | 1 | 95956363 | T | -0.0616 | 0.0138 | 0.007 | 19.91 | 8.12E-06 | LINC01761 |
| 12-hydroxyeicosatetraenoate (12-HETE) | | rs953628 | 3 | 98838914 | T | -0.0607 | 0.0134 | 0.007 | 20.51 | 5.74E-06 | RNU6-461P |

Abbreviations: Chr, chromosome; SE, standard error; SNP, single nucleotide polymorphism.
