# Supplementary material for: Causal links of human serum metabolites on the risk of prostate cancer: insights from genome-wide Mendelian randomization, single-cell RNA sequencing, and metabolic pathway analysis
Source: Front Endocrinol (Lausanne). 2024 Nov 12;15:1443330. doi: 10.3389/fendo.2024.1443330 (PMC11590024; doi:10.3389/fendo.2024.1443330)
Supplement: Supplementary file 1 [file DataSheet1.zip › Supplementary materials/Supplementary Table S6.docx]

**Table S6.** Significant metabolic pathways involved in the occurrence and progression of prostate cancer.

| **Trait** | **Metabolic pathway** | **Metabolites involved** | **P-value** | **Database** |
| --- | --- | --- | --- | --- |
| Prostate cancer | Valine, leucine and isoleucine biosynthesis | Valine | 0.026 | KEGG |
| Prostate cancer | Nicotinate and nicotinamide metabolism | N1-methyl-3-pyridone-4-carboxamide | 0.048 | KEGG |
